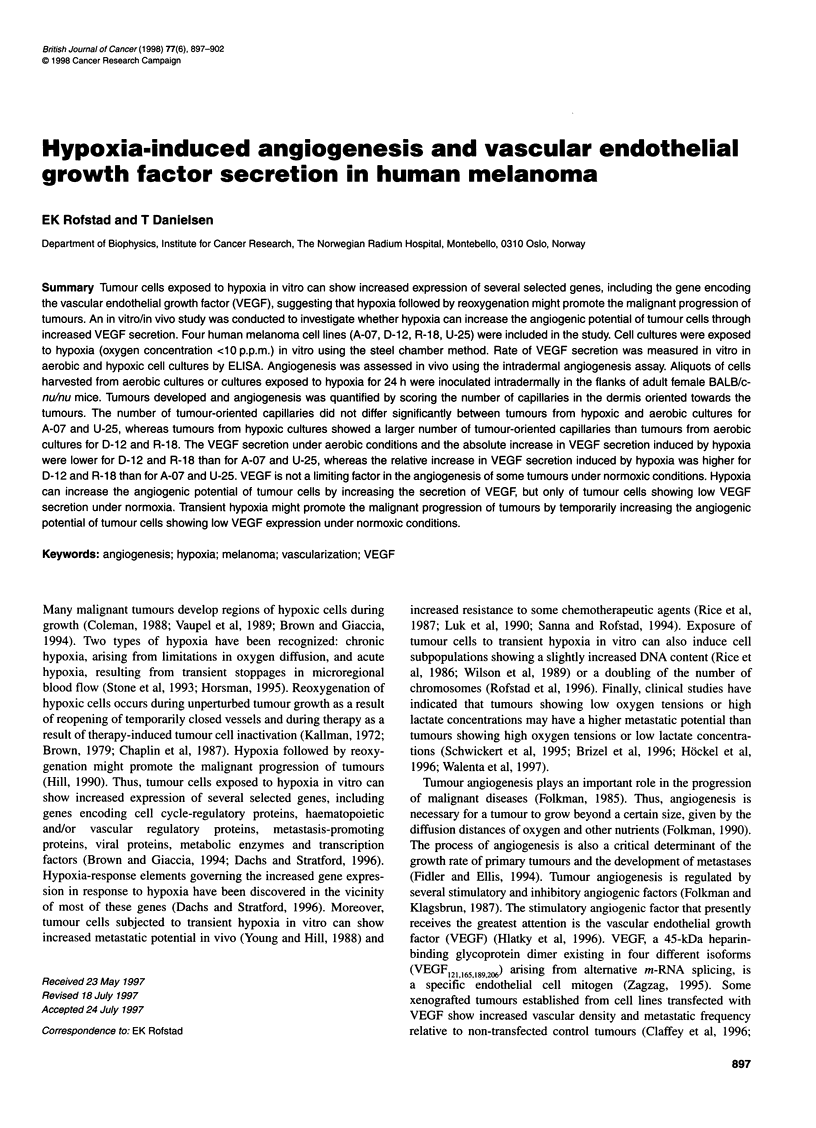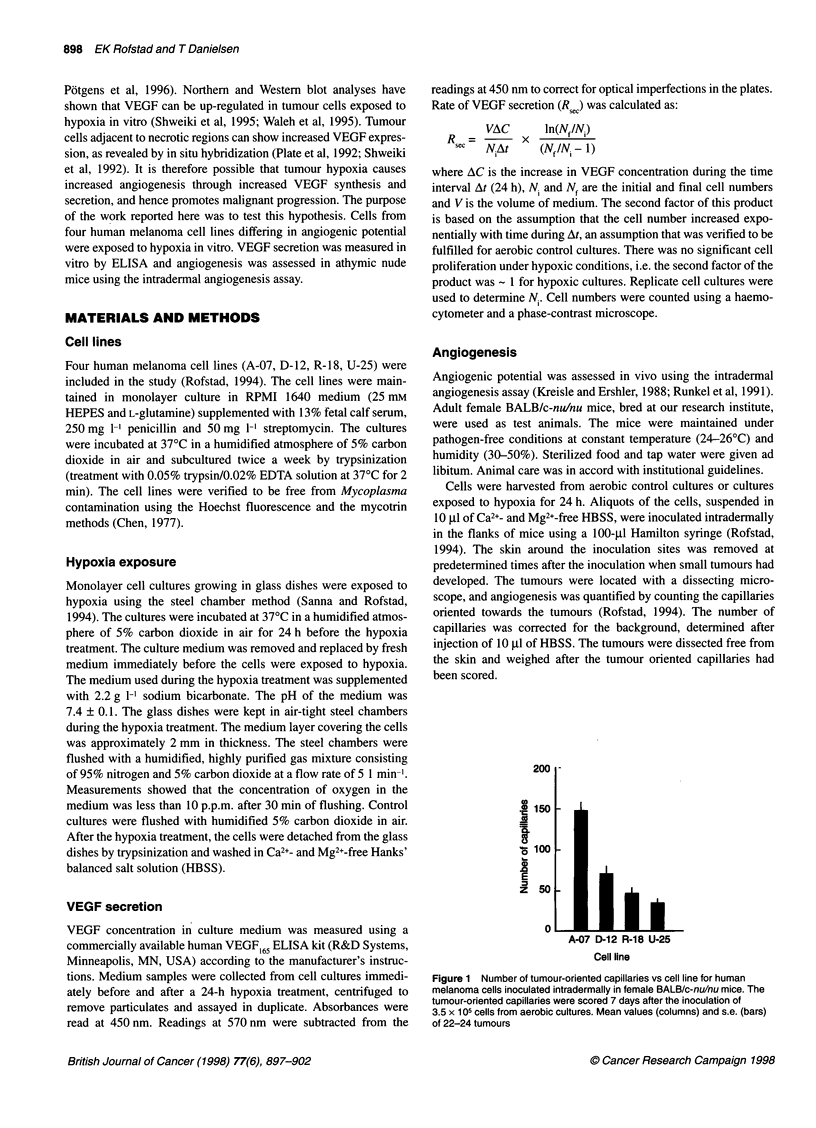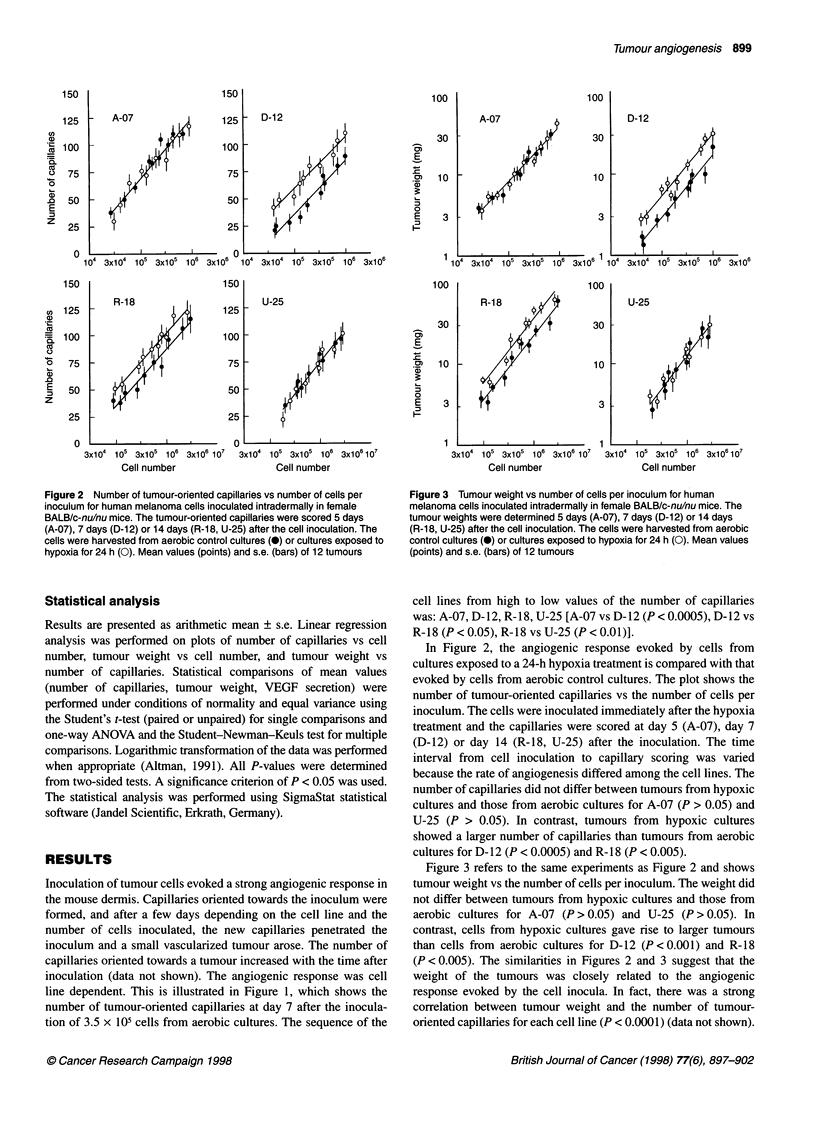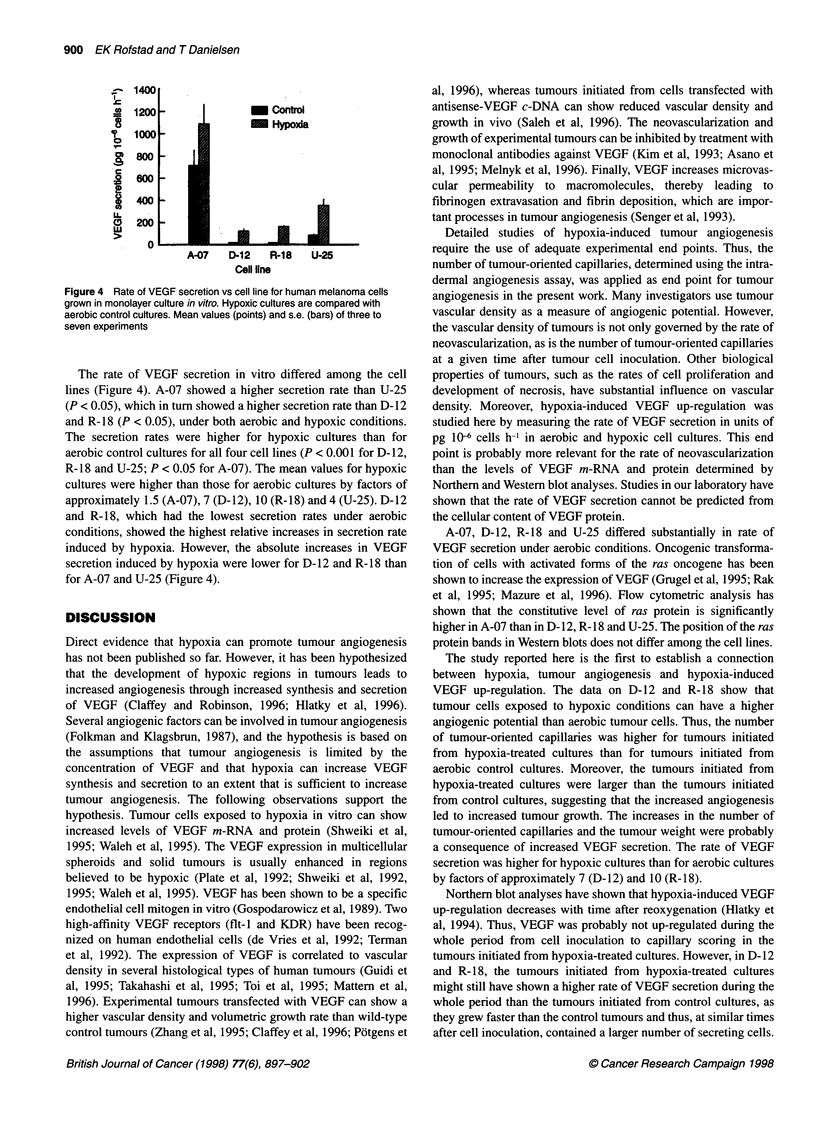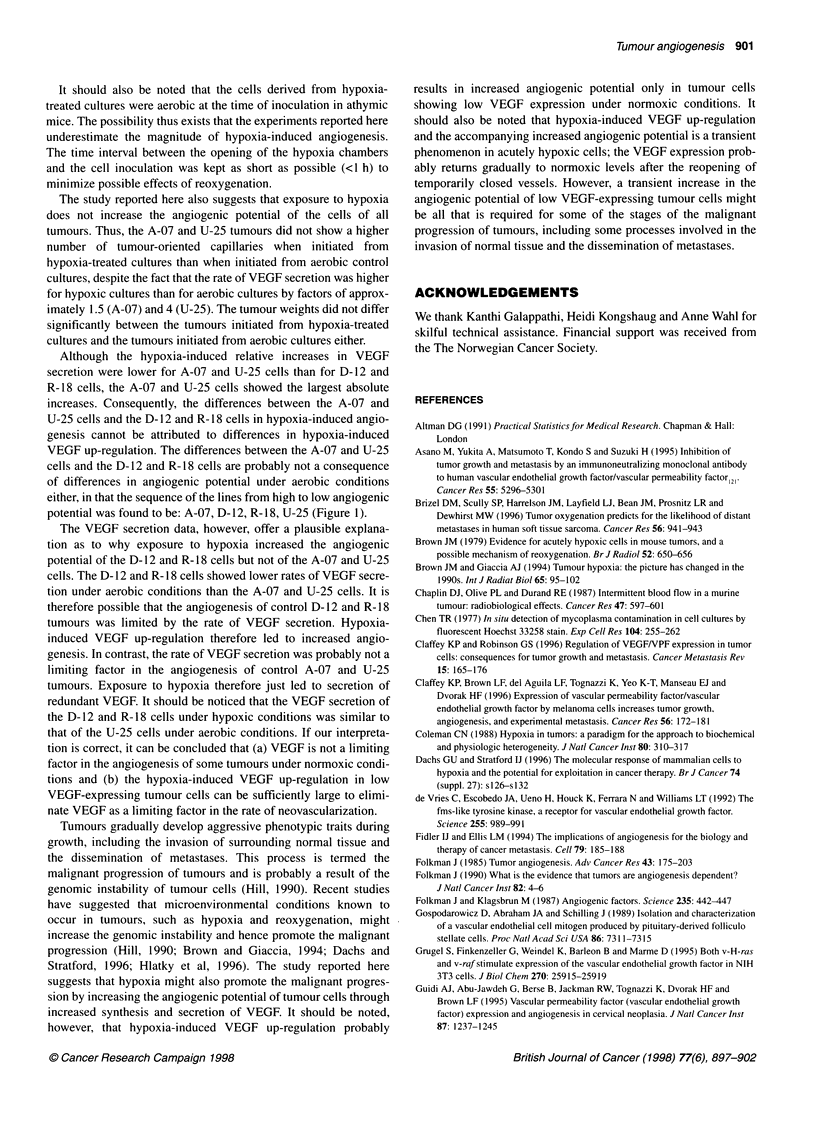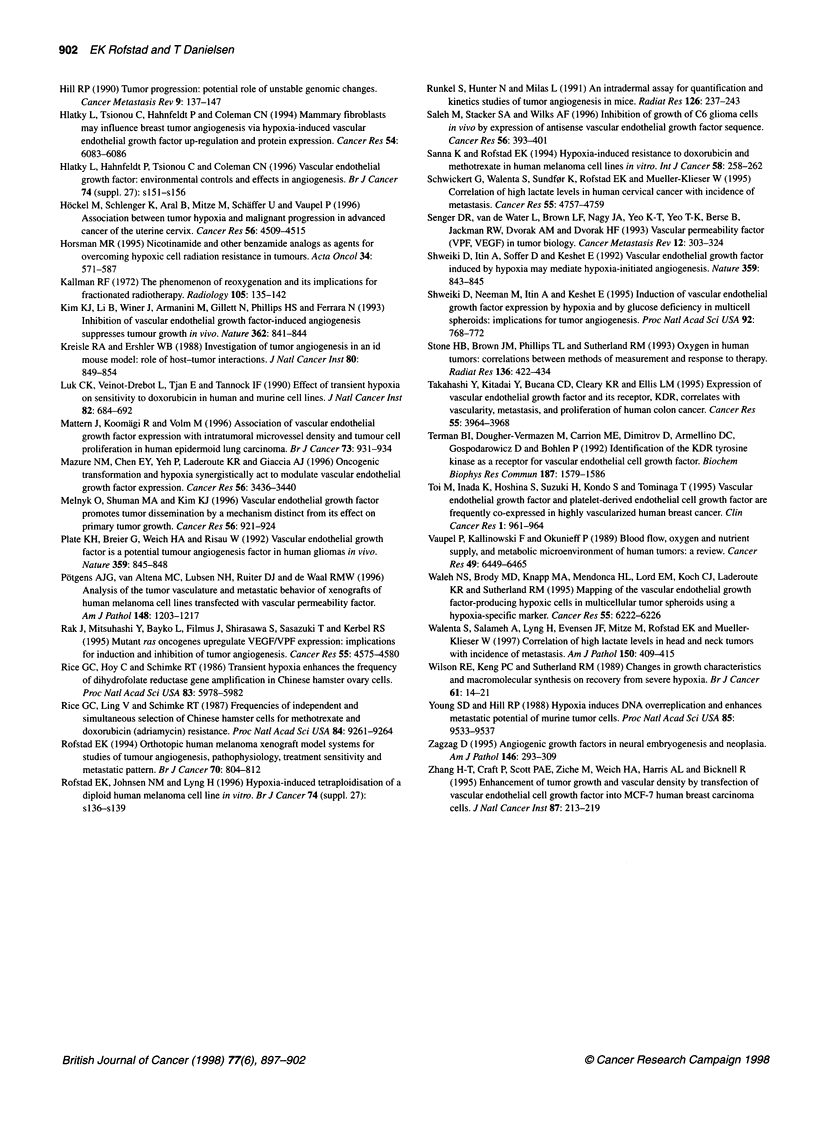# Hypoxia-induced angiogenesis and vascular endothelial growth factor secretion in human melanoma.

**DOI:** 10.1038/bjc.1998.148

**Published:** 1998-03

**Authors:** E. K. Rofstad, T. Danielsen

**Affiliations:** Department of Biophysics, Institute for Cancer Research, The Norwegian Radium Hospital, Montebello, Oslo.

## Abstract

Tumour cells exposed to hypoxia in vitro can show increased expression of several selected genes, including the gene encoding the vascular endothelial growth factor (VEGF), suggesting that hypoxia followed by reoxygenation might promote the malignant progression of tumours. An in vitro/in vivo study was conducted to investigate whether hypoxia can increase the angiogenic potential of tumour cells through increased VEGF secretion. Four human melanoma cell lines (A-07, D-12, R-18, U-25) were included in the study. Cell cultures were exposed to hypoxia (oxygen concentration <10 p.p.m.) in vitro using the steel chamber method. Rate of VEGF secretion was measured in vitro in aerobic and hypoxic cell cultures by ELISA. Angiogenesis was assessed in vivo using the intradermal angiogenesis assay. Aliquots of cells harvested from aerobic cultures or cultures exposed to hypoxia for 24 h were inoculated intradermally in the flanks of adult female BALB/c-nu/nu mice. Tumours developed and angiogenesis was quantified by scoring the number of capillaries in the dermis oriented towards the tumours. The number of tumour-oriented capillaries did not differ significantly between tumours from hypoxic and aerobic cultures for A-07 and U-25, whereas tumours from hypoxic cultures showed a larger number of tumour-oriented capillaries than tumours from aerobic cultures for D-12 and R-18. The VEGF secretion under aerobic conditions and the absolute increase in VEGF secretion induced by hypoxia were lower for D-12 and R-18 than for A-07 and U-25, whereas the relative increase in VEGF secretion induced by hypoxia was higher for D-12 and R-18 than for A-07 and U-25. VEGF is not a limiting factor in the angiogenesis of some tumours under normoxic conditions. Hypoxia can increase the angiogenic potential of tumour cells by increasing the secretion of VEGF, but only of tumour cells showing low VEGF secretion under normoxia. Transient hypoxia might promote the malignant progression of tumours by temporarily increasing the angiogenic potential of tumour cells showing low VEGF expression under normoxic conditions.


*British Journal of Cancer* (1998) **77**(6), 897–902
© 1998 Cancer Research Campaign

### Hypoxia-induced angiogenesis and vascular endothelial growth factor secretion in human melanoma

**EK Rofstad and T Danielsen**

Department of Biophysics, Institute for Cancer Research, The Norwegian Radium Hospital, Montebello, 0310 Oslo, Norway

**Summary** Tumour cells exposed to hypoxia in vitro can show increased expression of several selected genes, including the gene encoding the vascular endothelial growth factor (VEGF), suggesting that hypoxia followed by reoxygenation might promote the malignant progression of tumours. An in vitro/in vivo study was conducted to investigate whether hypoxia can increase the angiogenic potential of tumour cells through increased VEGF secretion. Four human melanoma cell lines (A-07, D-12, R-18, U-25) were included in the study. Cell cultures were exposed to hypoxia (oxygen concentration <10 p.p.m.) in vitro using the steel chamber method. Rate of VEGF secretion was measured in vitro in aerobic and hypoxic cell cultures by ELISA. Angiogenesis was assessed in vivo using the intradermal angiogenesis assay. Aliquots of cells harvested from aerobic cultures or cultures exposed to hypoxia for 24 h were inoculated intradermally in the flanks of adult female BALB/c-*nu/nu* mice. Tumours developed and angiogenesis was quantified by scoring the number of capillaries in the dermis oriented towards the tumours. The number of tumour-oriented capillaries did not differ significantly between tumours from hypoxic and aerobic cultures for A-07 and U-25, whereas tumours from hypoxic cultures showed a larger number of tumour-oriented capillaries than tumours from aerobic cultures for D-12 and R-18. The VEGF secretion under aerobic conditions and the absolute increase in VEGF secretion induced by hypoxia were lower for D-12 and R-18 than for A-07 and U-25, whereas the relative increase in VEGF secretion induced by hypoxia was higher for D-12 and R-18 than for A-07 and U-25. VEGF is not a limiting factor in the angiogenesis of some tumours under normoxic conditions. Hypoxia can increase the angiogenic potential of tumour cells by increasing the secretion of VEGF, but only of tumour cells showing low VEGF secretion under normoxia. Transient hypoxia might promote the malignant progression of tumours by temporarily increasing the angiogenic potential of tumour cells showing low VEGF expression under normoxic conditions.

**Keywords:** angiogenesis; hypoxia; melanoma; vascularization; VEGF

Many malignant tumours develop regions of hypoxic cells during growth (Coleman, 1988; Vaupel et al, 1989; Brown and Giaccia, 1994). Two types of hypoxia have been recognized: chronic hypoxia, arising from limitations in oxygen diffusion, and acute hypoxia, resulting from transient stoppages in microregional blood flow (Stone et al, 1993; Horsman, 1995). Reoxygenation of hypoxic cells occurs during unperturbed tumour growth as a result of reopening of temporarily closed vessels and during therapy as a result of therapy-induced tumour cell inactivation (Kallman, 1972; Brown, 1979; Chaplin et al, 1987). Hypoxia followed by reoxygenation might promote the malignant progression of tumours (Hill, 1990). Thus, tumour cells exposed to hypoxia in vitro can show increased expression of several selected genes, including genes encoding cell cycle-regulatory proteins, haematopoietic and/or vascular regulatory proteins, metastasis-promoting proteins, viral proteins, metabolic enzymes and transcription factors (Brown and Giaccia, 1994; Dachs and Stratford, 1996). Hypoxia-response elements governing the increased gene expression in response to hypoxia have been discovered in the vicinity of most of these genes (Dachs and Stratford, 1996). Moreover, tumour cells subjected to transient hypoxia in vitro can show increased metastatic potential in vivo (Young and Hill, 1988) and increased resistance to some chemotherapeutic agents (Rice et al, 1987; Luk et al, 1990; Sanna and Rofstad, 1994). Exposure of tumour cells to transient hypoxia in vitro can also induce cell subpopulations showing a slightly increased DNA content (Rice et al, 1986; Wilson et al, 1989) or a doubling of the number of chromosomes (Rofstad et al, 1996). Finally, clinical studies have indicated that tumours showing low oxygen tensions or high lactate concentrations may have a higher metastatic potential than tumours showing high oxygen tensions or low lactate concentrations (Schwickert et al, 1995; Brizel et al, 1996; Höckel et al, 1996; Walenta et al, 1997).

Tumour angiogenesis plays an important role in the progression of malignant diseases (Folkman, 1985). Thus, angiogenesis is necessary for a tumour to grow beyond a certain size, given by the diffusion distances of oxygen and other nutrients (Folkman, 1990). The process of angiogenesis is also a critical determinant of the growth rate of primary tumours and the development of metastases (Fidler and Ellis, 1994). Tumour angiogenesis is regulated by several stimulatory and inhibitory angiogenic factors (Folkman and Klagsbrun, 1987). The stimulatory angiogenic factor that presently receives the greatest attention is the vascular endothelial growth factor (VEGF) (Hlatky et al, 1996). VEGF, a 45-kDa heparin-binding glycoprotein dimer existing in four different isoforms ($VEGF_{121,165,189,206}$) arising from alternative *m*-RNA splicing, is a specific endothelial cell mitogen (Zagzag, 1995). Some xenografted tumours established from cell lines transfected with VEGF show increased vascular density and metastatic frequency relative to non-transfected control tumours (Claffey et al, 1996;

*Received 23 May 1997*
*Revised 18 July 1997*
*Accepted 24 July 1997*

*Correspondence to:* EK Rofstad

Pötgens et al, 1996). Northern and Western blot analyses have shown that VEGF can be up-regulated in tumour cells exposed to hypoxia in vitro (Shweiki et al, 1995; Waleh et al, 1995). Tumour cells adjacent to necrotic regions can show increased VEGF expression, as revealed by in situ hybridization (Plate et al, 1992; Shweiki et al, 1992). It is therefore possible that tumour hypoxia causes increased angiogenesis through increased VEGF synthesis and secretion, and hence promotes malignant progression. The purpose of the work reported here was to test this hypothesis. Cells from four human melanoma cell lines differing in angiogenic potential were exposed to hypoxia in vitro. VEGF secretion was measured in vitro by ELISA and angiogenesis was assessed in athymic nude mice using the intradermal angiogenesis assay.

### MATERIALS AND METHODS

### Cell lines

Four human melanoma cell lines (A-07, D-12, R-18, U-25) were included in the study (Rofstad, 1994). The cell lines were maintained in monolayer culture in RPMI 1640 medium (25 mM HEPES and L-glutamine) supplemented with 13% fetal calf serum, 250 mg $l^{-1}$ penicillin and 50 mg $l^{-1}$ streptomycin. The cultures were incubated at 37°C in a humidified atmosphere of 5% carbon dioxide in air and subcultured twice a week by trypsinization (treatment with 0.05% trypsin/0.02% EDTA solution at 37°C for 2 min). The cell lines were verified to be free from *Mycoplasma* contamination using the Hoechst fluorescence and the mycotrin methods (Chen, 1977).

### Hypoxia exposure

Monolayer cell cultures growing in glass dishes were exposed to hypoxia using the steel chamber method (Sanna and Rofstad, 1994). The cultures were incubated at 37°C in a humidified atmosphere of 5% carbon dioxide in air for 24 h before the hypoxia treatment. The culture medium was removed and replaced by fresh medium immediately before the cells were exposed to hypoxia. The medium used during the hypoxia treatment was supplemented with 2.2 g $l^{-1}$ sodium bicarbonate. The pH of the medium was 7.4 ± 0.1. The glass dishes were kept in air-tight steel chambers during the hypoxia treatment. The medium layer covering the cells was approximately 2 mm in thickness. The steel chambers were flushed with a humidified, highly purified gas mixture consisting of 95% nitrogen and 5% carbon dioxide at a flow rate of 5 l $min^{-1}$. Measurements showed that the concentration of oxygen in the medium was less than 10 p.p.m. after 30 min of flushing. Control cultures were flushed with humidified 5% carbon dioxide in air. After the hypoxia treatment, the cells were detached from the glass dishes by trypsinization and washed in $Ca^{2+}$- and $Mg^{2+}$-free Hanks' balanced salt solution (HBSS).

### VEGF secretion

VEGF concentration in culture medium was measured using a commercially available human $VEGF_{165}$ ELISA kit (R&D Systems, Minneapolis, MN, USA) according to the manufacturer's instructions. Medium samples were collected from cell cultures immediately before and after a 24-h hypoxia treatment, centrifuged to remove particulates and assayed in duplicate. Absorbances were read at 450 nm. Readings at 570 nm were subtracted from the readings at 450 nm to correct for optical imperfections in the plates. Rate of VEGF secretion ($R_{sec}$) was calculated as:

$$R_{sec} = \frac{V\Delta C}{N_i \Delta t} \times \frac{\ln(N_f/N_i)}{(N_f/N_i - 1)}$$

where $\Delta C$ is the increase in VEGF concentration during the time interval $\Delta t$ (24 h), $N_i$ and $N_f$ are the initial and final cell numbers and $V$ is the volume of medium. The second factor of this product is based on the assumption that the cell number increased exponentially with time during $\Delta t$, an assumption that was verified to be fulfilled for aerobic control cultures. There was no significant cell proliferation under hypoxic conditions, i.e. the second factor of the product was ~ 1 for hypoxic cultures. Replicate cell cultures were used to determine $N_i$. Cell numbers were counted using a haemocytometer and a phase-contrast microscope.

### Angiogenesis

Angiogenic potential was assessed in vivo using the intradermal angiogenesis assay (Kreisle and Ershler, 1988; Runkel et al, 1991). Adult female BALB/c-*nu/nu* mice, bred at our research institute, were used as test animals. The mice were maintained under pathogen-free conditions at constant temperature (24–26°C) and humidity (30–50%). Sterilized food and tap water were given ad libitum. Animal care was in accord with institutional guidelines.

Cells were harvested from aerobic control cultures or cultures exposed to hypoxia for 24 h. Aliquots of the cells, suspended in 10 µl of $Ca^{2+}$- and $Mg^{2+}$-free HBSS, were inoculated intradermally in the flanks of mice using a 100-µl Hamilton syringe (Rofstad, 1994). The skin around the inoculation sites was removed at predetermined times after the inoculation when small tumours had developed. The tumours were located with a dissecting microscope, and angiogenesis was quantified by counting the capillaries oriented towards the tumours (Rofstad, 1994). The number of capillaries was corrected for the background, determined after injection of 10 µl of HBSS. The tumours were dissected free from the skin and weighed after the tumour oriented capillaries had been scored.

**Figure 1** Number of tumour-oriented capillaries vs cell line for human melanoma cells inoculated intradermally in female BALB/c-*nu/nu* mice. The tumour-oriented capillaries were scored 7 days after the inoculation of $3.5 \times 10^5$ cells from aerobic cultures. Mean values (columns) and s.e. (bars) of 22–24 tumours

© Cancer Research Campaign 1998

**Figure 2** Number of tumour-oriented capillaries vs number of cells per inoculum for human melanoma cells inoculated intradermally in female BALB/c-*nu/nu* mice. The tumour-oriented capillaries were scored 5 days (A-07), 7 days (D-12) or 14 days (R-18, U-25) after the cell inoculation. The cells were harvested from aerobic control cultures (●) or cultures exposed to hypoxia for 24 h (○). Mean values (points) and s.e. (bars) of 12 tumours

**Figure 3** Tumour weight vs number of cells per inoculum for human melanoma cells inoculated intradermally in female BALB/c-*nu/nu* mice. The tumour weights were determined 5 days (A-07), 7 days (D-12) or 14 days (R-18, U-25) after the cell inoculation. The cells were harvested from aerobic control cultures (●) or cultures exposed to hypoxia for 24 h (○). Mean values (points) and s.e. (bars) of 12 tumours

### Statistical analysis

Results are presented as arithmetic mean ± s.e. Linear regression analysis was performed on plots of number of capillaries vs cell number, tumour weight vs cell number, and tumour weight vs number of capillaries. Statistical comparisons of mean values (number of capillaries, tumour weight, VEGF secretion) were performed under conditions of normality and equal variance using the Student's *t*-test (paired or unpaired) for single comparisons and one-way ANOVA and the Student–Newman–Keuls test for multiple comparisons. Logarithmic transformation of the data was performed when appropriate (Altman, 1991). All *P*-values were determined from two-sided tests. A significance criterion of $P < 0.05$ was used. The statistical analysis was performed using SigmaStat statistical software (Jandel Scientific, Erkrath, Germany).

### RESULTS

Inoculation of tumour cells evoked a strong angiogenic response in the mouse dermis. Capillaries oriented towards the inoculum were formed, and after a few days depending on the cell line and the number of cells inoculated, the new capillaries penetrated the inoculum and a small vascularized tumour arose. The number of capillaries oriented towards a tumour increased with the time after inoculation (data not shown). The angiogenic response was cell line dependent. This is illustrated in Figure 1, which shows the number of tumour-oriented capillaries at day 7 after the inoculation of $3.5 \times 10^5$ cells from aerobic cultures. The sequence of the cell lines from high to low values of the number of capillaries was: A-07, D-12, R-18, U-25 [A-07 vs D-12 ($P < 0.0005$), D-12 vs R-18 ($P < 0.05$), R-18 vs U-25 ($P < 0.01$)].

In Figure 2, the angiogenic response evoked by cells from cultures exposed to a 24-h hypoxia treatment is compared with that evoked by cells from aerobic control cultures. The plot shows the number of tumour-oriented capillaries vs the number of cells per inoculum. The cells were inoculated immediately after the hypoxia treatment and the capillaries were scored at day 5 (A-07), day 7 (D-12) or day 14 (R-18, U-25) after the inoculation. The time interval from cell inoculation to capillary scoring was varied because the rate of angiogenesis differed among the cell lines. The number of capillaries did not differ between tumours from hypoxic cultures and those from aerobic cultures for A-07 ($P > 0.05$) and U-25 ($P > 0.05$). In contrast, tumours from hypoxic cultures showed a larger number of capillaries than tumours from aerobic cultures for D-12 ($P < 0.0005$) and R-18 ($P < 0.005$).

Figure 3 refers to the same experiments as Figure 2 and shows tumour weight vs the number of cells per inoculum. The weight did not differ between tumours from hypoxic cultures and those from aerobic cultures for A-07 ($P > 0.05$) and U-25 ($P > 0.05$). In contrast, cells from hypoxic cultures gave rise to larger tumours than cells from aerobic cultures for D-12 ($P < 0.001$) and R-18 ($P < 0.005$). The similarities in Figures 2 and 3 suggest that the weight of the tumours was closely related to the angiogenic response evoked by the cell inocula. In fact, there was a strong correlation between tumour weight and the number of tumour-oriented capillaries for each cell line ($P < 0.0001$) (data not shown).

© *Cancer Research Campaign 1998* 

**Figure 4** Rate of VEGF secretion vs cell line for human melanoma cells grown in monolayer culture *in vitro*. Hypoxic cultures are compared with aerobic control cultures. Mean values (points) and s.e. (bars) of three to seven experiments

The rate of VEGF secretion in vitro differed among the cell lines (Figure 4). A-07 showed a higher secretion rate than U-25 ($P < 0.05$), which in turn showed a higher secretion rate than D-12 and R-18 ($P < 0.05$), under both aerobic and hypoxic conditions. The secretion rates were higher for hypoxic cultures than for aerobic control cultures for all four cell lines ($P < 0.001$ for D-12, R-18 and U-25; $P < 0.05$ for A-07). The mean values for hypoxic cultures were higher than those for aerobic cultures by factors of approximately 1.5 (A-07), 7 (D-12), 10 (R-18) and 4 (U-25). D-12 and R-18, which had the lowest secretion rates under aerobic conditions, showed the highest relative increases in secretion rate induced by hypoxia. However, the absolute increases in VEGF secretion induced by hypoxia were lower for D-12 and R-18 than for A-07 and U-25 (Figure 4).

### DISCUSSION

Direct evidence that hypoxia can promote tumour angiogenesis has not been published so far. However, it has been hypothesized that the development of hypoxic regions in tumours leads to increased angiogenesis through increased synthesis and secretion of VEGF (Claffey and Robinson, 1996; Hlatky et al, 1996). Several angiogenic factors can be involved in tumour angiogenesis (Folkman and Klagsbrun, 1987), and the hypothesis is based on the assumptions that tumour angiogenesis is limited by the concentration of VEGF and that hypoxia can increase VEGF synthesis and secretion to an extent that is sufficient to increase tumour angiogenesis. The following observations support the hypothesis. Tumour cells exposed to hypoxia in vitro can show increased levels of VEGF *m*-RNA and protein (Shweiki et al, 1995; Waleh et al, 1995). The VEGF expression in multicellular spheroids and solid tumours is usually enhanced in regions believed to be hypoxic (Plate et al, 1992; Shweiki et al, 1992, 1995; Waleh et al, 1995). VEGF has been shown to be a specific endothelial cell mitogen in vitro (Gospodarowicz et al, 1989). Two high-affinity VEGF receptors (flt-1 and KDR) have been recognized on human endothelial cells (de Vries et al, 1992; Terman et al, 1992). The expression of VEGF is correlated to vascular density in several histological types of human tumours (Guidi et al, 1995; Takahashi et al, 1995; Toi et al, 1995; Mattern et al, 1996). Experimental tumours transfected with VEGF can show a higher vascular density and volumetric growth rate than wild-type control tumours (Zhang et al, 1995; Claffey et al, 1996; Pötgens et al, 1996), whereas tumours initiated from cells transfected with antisense-VEGF *c*-DNA can show reduced vascular density and growth in vivo (Saleh et al, 1996). The neovascularization and growth of experimental tumours can be inhibited by treatment with monoclonal antibodies against VEGF (Kim et al, 1993; Asano et al, 1995; Melnyk et al, 1996). Finally, VEGF increases microvascular permeability to macromolecules, thereby leading to fibrinogen extravasation and fibrin deposition, which are important processes in tumour angiogenesis (Senger et al, 1993).

Detailed studies of hypoxia-induced tumour angiogenesis require the use of adequate experimental end points. Thus, the number of tumour-oriented capillaries, determined using the intradermal angiogenesis assay, was applied as end point for tumour angiogenesis in the present work. Many investigators use tumour vascular density as a measure of angiogenic potential. However, the vascular density of tumours is not only governed by the rate of neovascularization, as is the number of tumour-oriented capillaries at a given time after tumour cell inoculation. Other biological properties of tumours, such as the rates of cell proliferation and development of necrosis, have substantial influence on vascular density. Moreover, hypoxia-induced VEGF up-regulation was studied here by measuring the rate of VEGF secretion in units of pg $10^{-6}$ cells $h^{-1}$ in aerobic and hypoxic cell cultures. This end point is probably more relevant for the rate of neovascularization than the levels of VEGF *m*-RNA and protein determined by Northern and Western blot analyses. Studies in our laboratory have shown that the rate of VEGF secretion cannot be predicted from the cellular content of VEGF protein.

A-07, D-12, R-18 and U-25 differed substantially in rate of VEGF secretion under aerobic conditions. Oncogenic transformation of cells with activated forms of the *ras* oncogene has been shown to increase the expression of VEGF (Grugel et al, 1995; Rak et al, 1995; Mazure et al, 1996). Flow cytometric analysis has shown that the constitutive level of *ras* protein is significantly higher in A-07 than in D-12, R-18 and U-25. The position of the *ras* protein bands in Western blots does not differ among the cell lines.

The study reported here is the first to establish a connection between hypoxia, tumour angiogenesis and hypoxia-induced VEGF up-regulation. The data on D-12 and R-18 show that tumour cells exposed to hypoxic conditions can have a higher angiogenic potential than aerobic tumour cells. Thus, the number of tumour-oriented capillaries was higher for tumours initiated from hypoxia-treated cultures than for tumours initiated from aerobic control cultures. Moreover, the tumours initiated from hypoxia-treated cultures were larger than the tumours initiated from control cultures, suggesting that the increased angiogenesis led to increased tumour growth. The increases in the number of tumour-oriented capillaries and the tumour weight were probably a consequence of increased VEGF secretion. The rate of VEGF secretion was higher for hypoxic cultures than for aerobic cultures by factors of approximately 7 (D-12) and 10 (R-18).

Northern blot analyses have shown that hypoxia-induced VEGF up-regulation decreases with time after reoxygenation (Hlatky et al, 1994). Thus, VEGF was probably not up-regulated during the whole period from cell inoculation to capillary scoring in the tumours initiated from hypoxia-treated cultures. However, in D-12 and R-18, the tumours initiated from hypoxia-treated cultures might still have shown a higher rate of VEGF secretion during the whole period than the tumours initiated from control cultures, as they grew faster than the control tumours and thus, at similar times after cell inoculation, contained a larger number of secreting cells.

© Cancer Research Campaign 1998

It should also be noted that the cells derived from hypoxia-treated cultures were aerobic at the time of inoculation in athymic mice. The possibility thus exists that the experiments reported here underestimate the magnitude of hypoxia-induced angiogenesis. The time interval between the opening of the hypoxia chambers and the cell inoculation was kept as short as possible (<1 h) to minimize possible effects of reoxygenation.

The study reported here also suggests that exposure to hypoxia does not increase the angiogenic potential of the cells of all tumours. Thus, the A-07 and U-25 tumours did not show a higher number of tumour-oriented capillaries when initiated from hypoxia-treated cultures than when initiated from aerobic control cultures, despite the fact that the rate of VEGF secretion was higher for hypoxic cultures than for aerobic cultures by factors of approximately 1.5 (A-07) and 4 (U-25). The tumour weights did not differ significantly between the tumours initiated from hypoxia-treated cultures and the tumours initiated from aerobic cultures either.

Although the hypoxia-induced relative increases in VEGF secretion were lower for A-07 and U-25 cells than for D-12 and R-18 cells, the A-07 and U-25 cells showed the largest absolute increases. Consequently, the differences between the A-07 and U-25 cells and the D-12 and R-18 cells in hypoxia-induced angiogenesis cannot be attributed to differences in hypoxia-induced VEGF up-regulation. The differences between the A-07 and U-25 cells and the D-12 and R-18 cells are probably not a consequence of differences in angiogenic potential under aerobic conditions either, in that the sequence of the lines from high to low angiogenic potential was found to be: A-07, D-12, R-18, U-25 (Figure 1).

The VEGF secretion data, however, offer a plausible explanation as to why exposure to hypoxia increased the angiogenic potential of the D-12 and R-18 cells but not of the A-07 and U-25 cells. The D-12 and R-18 cells showed lower rates of VEGF secretion under aerobic conditions than the A-07 and U-25 cells. It is therefore possible that the angiogenesis of control D-12 and R-18 tumours was limited by the rate of VEGF secretion. Hypoxia-induced VEGF up-regulation therefore led to increased angiogenesis. In contrast, the rate of VEGF secretion was probably not a limiting factor in the angiogenesis of control A-07 and U-25 tumours. Exposure to hypoxia therefore just led to secretion of redundant VEGF. It should be noticed that the VEGF secretion of the D-12 and R-18 cells under hypoxic conditions was similar to that of the U-25 cells under aerobic conditions. If our interpretation is correct, it can be concluded that (a) VEGF is not a limiting factor in the angiogenesis of some tumours under normoxic conditions and (b) the hypoxia-induced VEGF up-regulation in low VEGF-expressing tumour cells can be sufficiently large to eliminate VEGF as a limiting factor in the rate of neovascularization.

Tumours gradually develop aggressive phenotypic traits during growth, including the invasion of surrounding normal tissue and the dissemination of metastases. This process is termed the malignant progression of tumours and is probably a result of the genomic instability of tumour cells (Hill, 1990). Recent studies have suggested that microenvironmental conditions known to occur in tumours, such as hypoxia and reoxygenation, might increase the genomic instability and hence promote the malignant progression (Hill, 1990; Brown and Giaccia, 1994; Dachs and Stratford, 1996; Hlatky et al, 1996). The study reported here suggests that hypoxia might also promote the malignant progression by increasing the angiogenic potential of tumour cells through increased synthesis and secretion of VEGF. It should be noted, however, that hypoxia-induced VEGF up-regulation probably results in increased angiogenic potential only in tumour cells showing low VEGF expression under normoxic conditions. It should also be noted that hypoxia-induced VEGF up-regulation and the accompanying increased angiogenic potential is a transient phenomenon in acutely hypoxic cells; the VEGF expression probably returns gradually to normoxic levels after the reopening of temporarily closed vessels. However, a transient increase in the angiogenic potential of low VEGF-expressing tumour cells might be all that is required for some of the stages of the malignant progression of tumours, including some processes involved in the invasion of normal tissue and the dissemination of metastases.

### ACKNOWLEDGEMENTS

We thank Kanthi Galappathi, Heidi Kongshaug and Anne Wahl for skilful technical assistance. Financial support was received from the The Norwegian Cancer Society.

### REFERENCES

Altman DG (1991) *Practical Statistics for Medical Research*. Chapman & Hall: London

Asano M, Yukita A, Matsumoto T, Kondo S and Suzuki H (1995) Inhibition of tumor growth and metastasis by an immunoneutralizing monoclonal antibody to human vascular endothelial growth factor/vascular permeability factor$_{121}$. *Cancer Res* **55**: 5296–5301

Brizel DM, Scully SP, Harrelson JM, Layfield LJ, Bean JM, Prosnitz LR and Dewhirst MW (1996) Tumor oxygenation predicts for the likelihood of distant metastases in human soft tissue sarcoma. *Cancer Res* **56**: 941–943

Brown JM (1979) Evidence for acutely hypoxic cells in mouse tumors, and a possible mechanism of reoxygenation. *Br J Radiol* **52**: 650–656

Brown JM and Giaccia AJ (1994) Tumour hypoxia: the picture has changed in the 1990s. *Int J Radiat Biol* **65**: 95–102

Chaplin DJ, Olive PL and Durand RE (1987) Intermittent blood flow in a murine tumour: radiobiological effects. *Cancer Res* **47**: 597–601

Chen TR (1977) *In situ* detection of mycoplasma contamination in cell cultures by fluorescent Hoechst 33258 stain. *Exp Cell Res* **104**: 255–262

Claffey KP and Robinson GS (1996) Regulation of VEGF/VPF expression in tumor cells: consequences for tumor growth and metastasis. *Cancer Metastasis Rev* **15**: 165–176

Claffey KP, Brown LF, del Aguila LF, Tognazzi K, Yeo K-T, Manseau EJ and Dvorak HF (1996) Expression of vascular permeability factor/vascular endothelial growth factor by melanoma cells increases tumor growth, angiogenesis, and experimental metastasis. *Cancer Res* **56**: 172–181

Coleman CN (1988) Hypoxia in tumors: a paradigm for the approach to biochemical and physiologic heterogeneity. *J Natl Cancer Inst* **80**: 310–317

Dachs GU and Stratford IJ (1996) The molecular response of mammalian cells to hypoxia and the potential for exploitation in cancer therapy. *Br J Cancer* **74** (suppl. 27): s126–s132

de Vries C, Escobedo JA, Ueno H, Houck K, Ferrara N and Williams LT (1992) The fms-like tyrosine kinase, a receptor for vascular endothelial growth factor. *Science* **255**: 989–991

Fidler IJ and Ellis LM (1994) The implications of angiogenesis for the biology and therapy of cancer metastasis. *Cell* **79**: 185–188

Folkman J (1985) Tumor angiogenesis. *Adv Cancer Res* **43**: 175–203

Folkman J (1990) What is the evidence that tumors are angiogenesis dependent? *J Natl Cancer Inst* **82**: 4–6

Folkman J and Klagsbrun M (1987) Angiogenic factors. *Science* **235**: 442–447

Gospodarowicz D, Abraham JA and Schilling J (1989) Isolation and characterization of a vascular endothelial cell mitogen produced by pituitary-derived folliculo stellate cells. *Proc Natl Acad Sci USA* **86**: 7311–7315

Grugel S, Finkenzeller G, Weindel K, Barleon B and Marme D (1995) Both v-H-*ras* and v-*raf* stimulate expression of the vascular endothelial growth factor in NIH 3T3 cells. *J Biol Chem* **270**: 25915–25919

Guidi AJ, Abu-Jawdeh G, Berse B, Jackman RW, Tognazzi K, Dvorak HF and Brown LF (1995) Vascular permeability factor (vascular endothelial growth factor) expression and angiogenesis in cervical neoplasia. *J Natl Cancer Inst* **87**: 1237–1245

© Cancer Research Campaign 1998 

Hill RP (1990) Tumor progression: potential role of unstable genomic changes. *Cancer Metastasis Rev* **9**: 137–147

Hlatky L, Tsionou C, Hahnfeldt P and Coleman CN (1994) Mammary fibroblasts may influence breast tumor angiogenesis via hypoxia-induced vascular endothelial growth factor up-regulation and protein expression. *Cancer Res* **54**: 6083–6086

Hlatky L, Hahnfeldt P, Tsionou C and Coleman CN (1996) Vascular endothelial growth factor: environmental controls and effects in angiogenesis. *Br J Cancer* **74** (suppl. 27): s151–s156

Höckel M, Schlenger K, Aral B, Mitze M, Schäffer U and Vaupel P (1996) Association between tumor hypoxia and malignant progression in advanced cancer of the uterine cervix. *Cancer Res* **56**: 4509–4515

Horsman MR (1995) Nicotinamide and other benzamide analogs as agents for overcoming hypoxic cell radiation resistance in tumours. *Acta Oncol* **34**: 571–587

Kallman RF (1972) The phenomenon of reoxygenation and its implications for fractionated radiotherapy. *Radiology* **105**: 135–142

Kim KJ, Li B, Winer J, Armanini M, Gillett N, Phillips HS and Ferrara N (1993) Inhibition of vascular endothelial growth factor-induced angiogenesis suppresses tumour growth *in vivo*. *Nature* **362**: 841–844

Kreisle RA and Ershler WB (1988) Investigation of tumor angiogenesis in an id mouse model: role of host–tumor interactions. *J Natl Cancer Inst* **80**: 849–854

Luk CK, Veinot-Drebot L, Tjan E and Tannock IF (1990) Effect of transient hypoxia on sensitivity to doxorubicin in human and murine cell lines. *J Natl Cancer Inst* **82**: 684–692

Mattern J, Koomägi R and Volm M (1996) Association of vascular endothelial growth factor expression with intratumoral microvessel density and tumour cell proliferation in human epidermoid lung carcinoma. *Br J Cancer* **73**: 931–934

Mazure NM, Chen EY, Yeh P, Laderoute KR and Giaccia AJ (1996) Oncogenic transformation and hypoxia synergistically act to modulate vascular endothelial growth factor expression. *Cancer Res* **56**: 3436–3440

Melnyk O, Shuman MA and Kim KJ (1996) Vascular endothelial growth factor promotes tumor dissemination by a mechanism distinct from its effect on primary tumor growth. *Cancer Res* **56**: 921–924

Plate KH, Breier G, Weich HA and Risau W (1992) Vascular endothelial growth factor is a potential tumour angiogenesis factor in human gliomas *in vivo*. *Nature* **359**: 845–848

Pötgens AJG, van Altena MC, Lubsen NH, Ruiter DJ and de Waal RMW (1996) Analysis of the tumor vasculature and metastatic behavior of xenografts of human melanoma cell lines transfected with vascular permeability factor. *Am J Pathol* **148**: 1203–1217

Rak J, Mitsuhashi Y, Bayko L, Filmus J, Shirasawa S, Sasazuki T and Kerbel RS (1995) Mutant *ras* oncogenes upregulate VEGF/VPF expression: implications for induction and inhibition of tumor angiogenesis. *Cancer Res* **55**: 4575–4580

Rice GC, Hoy C and Schimke RT (1986) Transient hypoxia enhances the frequency of dihydrofolate reductase gene amplification in Chinese hamster ovary cells. *Proc Natl Acad Sci USA* **83**: 5978–5982

Rice GC, Ling V and Schimke RT (1987) Frequencies of independent and simultaneous selection of Chinese hamster cells for methotrexate and doxorubicin (adriamycin) resistance. *Proc Natl Acad Sci USA* **84**: 9261–9264

Rofstad EK (1994) Orthotopic human melanoma xenograft model systems for studies of tumour angiogenesis, pathophysiology, treatment sensitivity and metastatic pattern. *Br J Cancer* **70**: 804–812

Rofstad EK, Johnsen NM and Lyng H (1996) Hypoxia-induced tetraploidisation of a diploid human melanoma cell line *in vitro*. *Br J Cancer* **74** (suppl. 27): s136–s139

Runkel S, Hunter N and Milas L (1991) An intradermal assay for quantification and kinetics studies of tumor angiogenesis in mice. *Radiat Res* **126**: 237–243

Saleh M, Stacker SA and Wilks AF (1996) Inhibition of growth of C6 glioma cells *in vivo* by expression of antisense vascular endothelial growth factor sequence. *Cancer Res* **56**: 393–401

Sanna K and Rofstad EK (1994) Hypoxia-induced resistance to doxorubicin and methotrexate in human melanoma cell lines *in vitro*. *Int J Cancer* **58**: 258–262

Schwickert G, Walenta S, Sundfør K, Rofstad EK and Mueller-Klieser W (1995) Correlation of high lactate levels in human cervical cancer with incidence of metastasis. *Cancer Res* **55**: 4757–4759

Senger DR, van de Water L, Brown LF, Nagy JA, Yeo K-T, Yeo T-K, Berse B, Jackman RW, Dvorak AM and Dvorak HF (1993) Vascular permeability factor (VPF, VEGF) in tumor biology. *Cancer Metastasis Rev* **12**: 303–324

Shweiki D, Itin A, Soffer D and Keshet E (1992) Vascular endothelial growth factor induced by hypoxia may mediate hypoxia-initiated angiogenesis. *Nature* **359**: 843–845

Shweiki D, Neeman M, Itin A and Keshet E (1995) Induction of vascular endothelial growth factor expression by hypoxia and by glucose deficiency in multicell spheroids: implications for tumor angiogenesis. *Proc Natl Acad Sci USA* **92**: 768–772

Stone HB, Brown JM, Phillips TL and Sutherland RM (1993) Oxygen in human tumors: correlations between methods of measurement and response to therapy. *Radiat Res* **136**: 422–434

Takahashi Y, Kitadai Y, Bucana CD, Cleary KR and Ellis LM (1995) Expression of vascular endothelial growth factor and its receptor, KDR, correlates with vascularity, metastasis, and proliferation of human colon cancer. *Cancer Res* **55**: 3964–3968

Terman BI, Dougher-Vermazen M, Carrion ME, Dimitrov D, Armellino DC, Gospodarowicz D and Bohlen P (1992) Identification of the KDR tyrosine kinase as a receptor for vascular endothelial cell growth factor. *Biochem Biophys Res Commun* **187**: 1579–1586

Toi M, Inada K, Hoshina S, Suzuki H, Kondo S and Tominaga T (1995) Vascular endothelial growth factor and platelet-derived endothelial cell growth factor are frequently co-expressed in highly vascularized human breast cancer. *Clin Cancer Res* **1**: 961–964

Vaupel P, Kallinowski F and Okunieff P (1989) Blood flow, oxygen and nutrient supply, and metabolic microenvironment of human tumors: a review. *Cancer Res* **49**: 6449–6465

Waleh NS, Brody MD, Knapp MA, Mendonca HL, Lord EM, Koch CJ, Laderoute KR and Sutherland RM (1995) Mapping of the vascular endothelial growth factor-producing hypoxic cells in multicellular tumor spheroids using a hypoxia-specific marker. *Cancer Res* **55**: 6222–6226

Walenta S, Salameh A, Lyng H, Evensen JF, Mitze M, Rofstad EK and Mueller-Klieser W (1997) Correlation of high lactate levels in head and neck tumors with incidence of metastasis. *Am J Pathol* **150**: 409–415

Wilson RE, Keng PC and Sutherland RM (1989) Changes in growth characteristics and macromolecular synthesis on recovery from severe hypoxia. *Br J Cancer* **61**: 14–21

Young SD and Hill RP (1988) Hypoxia induces DNA overreplication and enhances metastatic potential of murine tumor cells. *Proc Natl Acad Sci USA* **85**: 9533–9537

Zagzag D (1995) Angiogenic growth factors in neural embryogenesis and neoplasia. *Am J Pathol* **146**: 293–309

Zhang H-T, Craft P, Scott PAE, Ziche M, Weich HA, Harris AL and Bicknell R (1995) Enhancement of tumor growth and vascular density by transfection of vascular endothelial cell growth factor into MCF-7 human breast carcinoma cells. *J Natl Cancer Inst* **87**: 213–219

 © Cancer Research Campaign 1998